# Elucidation of Conduction Mechanism in Graphene Nanoplatelets (GNPs)/Cement Composite Using Dielectric Spectroscopy

**DOI:** 10.3390/ma13020275

**Published:** 2020-01-08

**Authors:** Guido Goracci, Jorge S. Dolado

**Affiliations:** 1 BASKRETE-Euskampus Fundazioa, Ed. Rectorado Barrio Sarriena s/n, 48940 Leioa, Spain; 2 Centro de Física de Materiales, (CSIC-UPV/EHU)-Material Physics Centre (MPC), Paseo Manuel de Lardizabal 5, 20018 San Sebastián, Spain; jorge_dolado002@ehu.eus; 3 Donostia International Physics Center (DIPC), Paseo Manuel Lardizábal 4, 20018 Donostia-San Sebastián, Spain

**Keywords:** electrical conductive concrete, multifunctional composite, conductive filler, graphene nanoplatelets, dielectric properties, electric properties

## Abstract

Understanding the mechanisms that govern the conductive properties of multifunctional cement-materials is fundamental for the development of the new applications proposed to enhance the energy efficiency, safety and structural properties of smart buildings and infrastructures. Many fillers have been suggested to increase the electrical conduction in concretes; however, the processes involved are still not entirely known. In the present work, we investigated the effect of graphene nanoplatelets (1 wt% on the electrical properties of cement composites (OPC/GNPs). We found a decrease of the bulk resistivity in the composite associated to the enhancement of the charge transport properties in the sample. Moreover, the study of the dielectric properties suggests that the main contribution to conduction is given by water diffusion through the porous network resulting in ion conductivity. Finally, the results support that the increase of direct current in OPC/GNPs is due to pore refinement induced by graphene nanoplatelets.

## 1. Introduction

In recent years, the interest on the smart city concept to promote environmental sustainability through the implementation of new technologies has shown a large growth. New construction technologies have been investigated to enhance the energy efficiency, safety, and structural performance of buildings and infrastructure. In this framework, the development of multifunctional cement-based materials has a key role. Indeed, a strong effort is needed to design innovative concretes that could serve as structural material with tailored functional behavior to meet specific requirements.

Multifunctional cement-based materials have been proposed for several applications [1]. Regarding the infrastructure, much interest has been devoted to snow melting and de-icing systems with conductive concrete composites as heating elements [2,3,4], cathodic protection of steel reinforcement concrete to prevent corrosion damages [5,6,7] and traffic sensors with conductive concrete [8]. Cement-based composites have been proposed for grounding systems [9,10] as well as electromagnetic wave shielding [11,12]. Regarding the development of smart buildings, structural health monitoring systems are fundamental for the modern structures and concrete composite sensors are a good candidate due to the intrinsic compatibility with the cement matrix [1]. Finally, multifunctional cement-based materials have been suggested for the development of structural supercapacitors [13,14,15].

All the applications mentioned above base their efficiency on the conductive properties of the structural material. However, it is well known that concrete is characterized by an insulating behavior. To overcome such a drawback, different conductive fillers have been indicated as good aggregates to achieve the design of conductive concrete [1,16]. Metal conductive admixtures have been proposed with the addition of steel fibers and micro fibers [17,18,19] and steel shaving [2]. Among the carbon admixtures, graphite [20,21,22], carbon fibers [3,18,19,23,24,25,26] and graphene [27,28] have been investigated for electrical conductive concretes. Finally, carbon nanomaterials gathered a lot of attention as they have, beyond high electrical conductivity, unique physical properties [16,29]. Among carbon nanomaterials employed in concrete composites we mention carbon black [30,31], carbon nanotubes [32,33,34,35] and nanofibers [36,37,38,39,40], as well as graphene nanoplatelets [41,42,43,44,45].

Performance of electrical conductive concrete depends clearly on the nature and amount of the filler. However, it has been demonstrated that water as well plays a crucial role in the conduction mechanism. Indeed, electrical resistivity depends on aging due to changes of pore water amount [18,40] and an ionic conduction in wet concretes has been observed due to the free water molecules [46]. Nonetheless, the conductive mechanism in cement-based materials is still not completely understood. Moreover, the studies on the effect of fillers on electrical properties focused the attention mainly on the formation of a conduction path.

With the aim of elucidating the processes involved in conduction in cement-based materials and, in particular, of investigating the role of water molecules and the indirect impact of the addition of fillers, we studied graphene nanoplatelets/cement composite by means of dielectric spectroscopy technique at different temperatures. Such a technique, commonly used to study conductivity in ceramic materials [47,48,49,50,51,52] and dielectric and electrical properties of porous systems [53,54,55,56,57,58,59,60] and polymer composite [61,62,63,64,65,66,67,68], is a very suitable tool due to its unique properties. In fact, due to its large frequency range, it is possible to investigate the impedance and dielectric response of the material on different time scale and, therefore, to obtain information on the different processes involved in the ionic and electronic conduction phenomena. First, due to the strong response of dielectric spectroscopy to water amount, thermal gravimetric analysis results are shown. Therefore, the impedance response of the specimens is examined to understand how graphene nanoplatelets affect the electrical properties. Finally, the dielectric response of the system is discussed to reveal the conduction mechanism and the role of the filler.

## 2. Materials and Methods

In this study, two samples were prepared: OPC paste (as reference) and OPC/Graphene nanoplatelets (GNPs) composite. To focus the attention on ion conduction, the porosity of the system was increased by using a water-to-cement ratio of w/c = 0.6 and curing the samples during seven days. Moreover we added only 1 wt% of GNPs to keep the sample below the percolation threshold suggested by literature [41] to minimize the electrical conduction that may contributes when a complete conductive path is formed. The cement used was CEM II/A-LL 42.5 R and GPL from GrapheneTech (Zaragoza, Spain) was used as filler. This product presents a specific surface area around 200 m^2^ g^−1^, lateral size between 500–1000 nm and a carbon content above 97%. For sample preparation, first powders were mixed using a mechanical blender at low speed (350 rpm) for 1 min to obtain a uniform dispersion of GNPs in the OPC powder. Afterwards, ultrapure water was added and the solution was mixed at 750 rpm. Both sets were cast in cylindrical silicone molds with d = 4 cm and sealed. After 24 h, specimens were demolded and cured in water for 7 days. Finally, the cylinders were crashed into fine powder and kept overnight in desiccator with silica gel before measured.

Thermal gravimetric analyses were carried out using a TGA-500 (TA Instruments, New Castle, DE, USA) to investigate the water amount in the samples and verify if any phase transformation occurs when GNPs are added. All the measurements were conducted under high-purity nitrogen flow over a temperature of 303–1173 °C with a ramp rate of 5 K/min.

A broadband dielectric spectrometer, Novocontrol Alpha-A, (Novocontrol, Montabaur, Germany) was used to measure the complex dielectric permittivity, defined as $\varepsilon^{*}\left(\omega \right)=C^{*}\left(\omega \right)/C_{0}=1/\left(i\omega Z^{*}\left(\omega \right)C_{0}\right)\;=\;\varepsilon^{\prime }\left(\omega \right)-i\varepsilon^{\prime \prime }\left(\omega \right)$ where *C*_0_ is the capacitance of the free space, C* is the complex capacitance function, Z* is the complex impedance and ω=2πf the angular frequency with f the applied electric field frequency. Data were collected over a broad frequency range, from 10^−2^ to 10^6^ Hz. Samples were prepared by placing the sample powder between two parallel gold-plated electrodes of a diameter of d = 30 mm and thickness of about 0.7 mm. First, the sample was kept at room temperature inside the spectrometer during 10 min to overcome humidity signal. Therefore, isothermal scans were performed on heating every 5 degrees over the temperature range of 290–310 K. Temperature was controlled by a nitrogen gas flow with stability better than ±0.1 K.

## 3. Results and Discussion

### 3.1. Thermal Gravimetric Analysis

**Figure 1:**
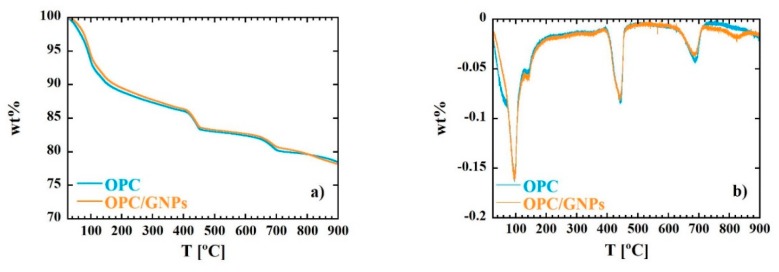
Thermal Gravimetric (TG) (**a**) and Differential Thermal Gravimetric (DTG) (**b**) curves for OPC and OPC/GNPs samples.

Thermal Gravimetric Analysis (TGA) and Differential Thermal Gravimetric (DTG) (TA instruments, New Castle, DE, USA) measurements allow to identify the different water population in the sample. These data are deeply relevant for the interpretation of the dielectric response of the material. TGA and DTG curves of OPC and OPC/GNPs samples are shown in Figure 1. Clearly, both TGA and DTG measurements share similar temperature dependence behavior. The first peak at ~98 °C in DTG curve is associated with evaporable water in C-S-H gel and ettringite [69,70]. At ~140 °C we observe a further decrease in weight than can be related to gypsum or amorphous carbon illuminate hydrate decompositions [71]. Moreover, a stiff decrease is observed in TGA curve in the range between 390 °C–460 °C. Such event is associated to the dehydroxylation of Ca(OH)_2_ [72]. Finally, the peak at around ~680 °C is related to the decarbonation, together with possible solid-solid phase transformations [69].

In OPC/GNPs sample, a further peak in DTG curve is observed at ~825 °C. In Table 1, the weight percent loss corresponding to water and portlandite is shown. Both samples are characterized by a similar amount of free water (~7 wt%) at a temperature lower than 105 °C) and physical bound water of hydrates (~7.5 wt%). Moreover, OPC and OPC/GNPs specimens contains the same amount of Portlandite (15 wt%).

### 3.2. Impedance Response

**Figure 2:**
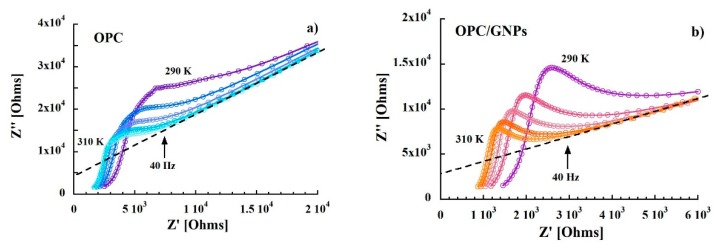
Nyquist plot of the impedance of OPC (**a**) and OPC/GNPs (**b**). Dashed line is the extrapolation to high frequencies of the cement-electrode interface contribution.

**Figure 3:**
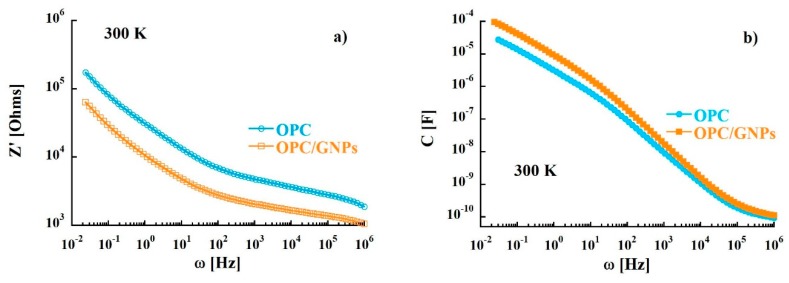
(**a**) Dependence of the real part of the complex impedance function as a function of frequency. (**b**) Frequency dependence of capacitance at 300 K.

**Figure 4:**
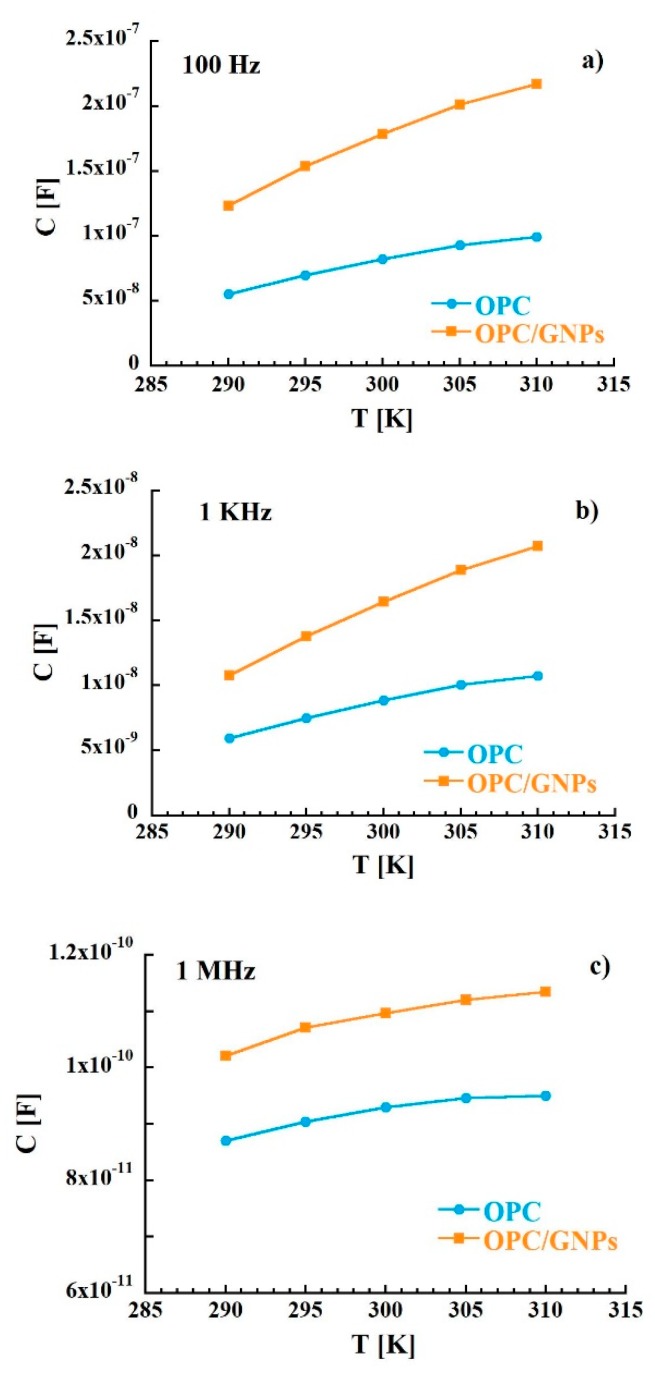
Temperature dependence of the capacitance as at 100 Hz (**a**), 1 KHz (**b**) and 1 MHz (**c**).

The complex impedance function is defined as:

(1) $$Z^{*}=Z^{\prime }+{\mathrm{iZ}}^{\prime \prime }$$

where the real part and the imaginary part are defined as Z′ = R and Z″ = 1/ωC, with R and C resistance and capacitance respectively. The plot in the complex Z″-Z′ plane, called Nyquist plot (Z″ vs. Z′), in the temperature range investigated (290–310 K) is shown in Figure 2. This representation allows to separate bulk properties from electrode polarization [73,74]. In the OPC sample, in the low frequency region (right side of the figure), a sloped line that can be related to cement-electrode interface contribution is observed [75,76,77]. At around 40 Hz, the line starts to diverge to a broad and asymmetric semi-circle corresponding to an overlap of polarization mechanisms in the bulk. From the intersection between interface and polarization contributions the bulk resistance value of 8.9 KΩ at 300 K was extracted. When graphene nanoplatelets are added to the cement paste some relevant changes in the Nyquist plot are observed. In fact, a stretching of the semi-arcs for frequencies higher than 40 Hz was clearly noticed and the bulk resistance at room temperature decreased to 3.9 KΩ indicating an enhance of charge transport in the composite sample. Such behavior is confirmed when the real part of the impedance of the reference is compared to that of the composite. In fact, the resistance of the OPC samples is, at low frequencies, almost three times higher than that measured for OPC/GNPs (see Figure 3a) and, even though such difference decreases with frequency, Z′ values of OPC are still two times larger than those observed in OPC/GNPs for f > 1 KHz. This effect has been already observed in alkali activated slag (AAS) composites with graphene and carbon nanotubes addition and it was related to the creation of conductive paths resulting in a reduction of electrical resistance [78,79,80]. However, due to the small amount of GNPs in our composite, a continuous conductive path is not formed as demonstrated by Bai et al. [41]. In Figure 3b, the capacitance of OPC and OPC/GNPs at 300 K is compared. In the low frequency region, the addition of graphene nanoplatelets increases the electrical capacitance of three times. However, as the frequency increased, the difference between the two samples almost disappeared. Finally, we observed an increment of C values as a function of temperature (Figure 4): Such effect appears stronger for f ≤ 1 KHz and, in particular, in the composite sample. Summarizing, an enhancement of electrical capacitance and charge transport properties is observed when 1 wt% of GNPs is added to the cement paste. As such an amount of filler is not sufficient to create a complete conductive path, a deeper analysis must be carried out to clarify how the fillers lead to the electrical improvements in the composite.

### 3.3. Dielectric Response

Dielectric Spectroscopy allows us to investigate molecules dynamics, charge transport and interface interactions through the study of dipole reorientations when an alternative electric field is applied. In fact, depending on the temperature and frequency range, different dipole relaxations are observed, reflecting the distinct nature of the processes and interactions with the environment.

**Figure 5:**
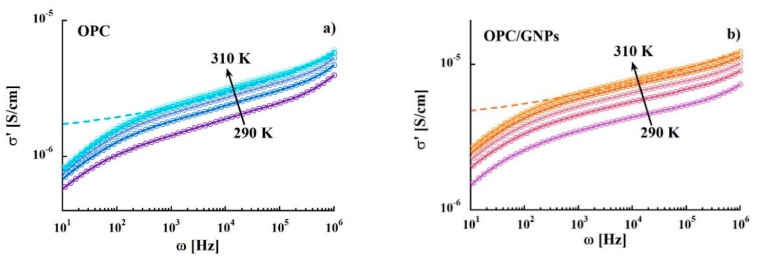
Electrical conductivity $\sigma^{\prime }\left(\omega \right)$ as a function of frequency in the temperature range 290–310 K of OPC (**a**) and OPC/GNPs (**b**).

Through the characterization of the energy activations and characteristic relaxation times the information on the origin of the processes can be extracted. Moreover, the complex conductivity function can be calculated from the dielectric loss according to the relation $\sigma^{*}\left(\omega \right)=\sigma^{\prime }\left(\omega \right)+i\sigma^{\prime \prime }\left(\omega \right)=i\omega \varepsilon^{*}\left(\omega \right)$ and, hence, it is possible to investigate the mechanisms that mark the electrical properties of the system. Figure 5 shows the variation of the real part of the complex conductivity $\sigma^{\prime }\left(\omega \right)$ in the temperature range 290–310 K. The presence of GNPs leads to an increase of $\sigma^{\prime }\left(\omega \right)$ values in the whole frequency range. In both samples, the same frequency pattern is observed: (1) frequency dependent conductivity at high frequencies (f > 10^5^ Hz) indicating an alternating current (ac) dominating contribution (2) an almost flat region related to the direct current (dc) (10^1^ to 10^5^ Hz) (3) a drastic drop at frequencies <100 Hz due to electrode polarization effects. Data were analyzed by using:

(2) $$\sigma^{\prime }\left(\omega \right)=\sigma_{DC}+A\omega^{n}$$

where $\sigma_{DC}$ is the dc conductivity, *A* is the pre-exponential factor and *n* is the exponential factor with values between 0 and 1 [81]. The resulting parameters are listed in Table 2. As expected, $\sigma_{DC}$ increases with temperature in both reference sample and composite. Moreover, the n parameter values of 0.17 tend low compared to those obtained for ionic conductors, 0.5 < *n* < 1, [82] and they are independent from both temperature and graphene nanoplatelets addition. In contrast, the values of $\sigma_{DC}$ are affected by GNPs: indeed, at room temperature they increase 4 times with respect to those measured in the reference sample at room temperature, and this effect grows with temperature.

**Figure 6:**
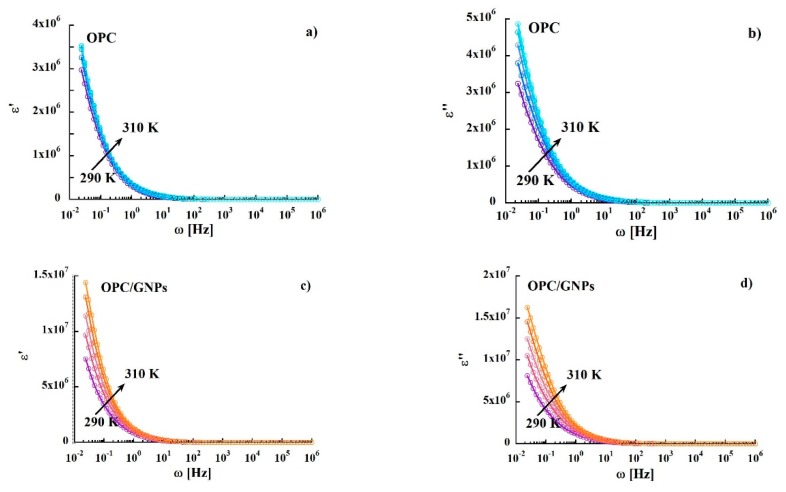
Frequency dependence of real and imaginary part of permittivity in OPC, (**a**,**b**), and in OPC/GNPs, (**c**,**d**), in the temperature range 290–310 K.

Additionally, the temperature dependence of conductivity was analyzed considering the dc conductivity as a thermally activated process with activation energy calculated according to the Arrhenius relation [83]:

(3) $$\sigma_{DC}=\sigma_{0}\exp(-\frac{E_{A}}{k_{B}T})$$

where *σ*_0_ is the pre-exponential factor associated with the charge carrier mobility and density of states, *E_A_* is the activation energy, *k_B_* is the Boltzmann constant and *T* is the temperature. The obtained activation energies, shown in Table 3, are low compared to those found for hopping conductivity (typically about 0.7–0.9 eV) [84]. Finally, we observed a decrease of the activation energy as we add graphene nanoplatelets. Figure 6 compares the variation of the real and imaginary part of the complex permittivity function of the samples with frequency at different temperatures. Regarding the real part ε′ (Figure 6a,c) a strong dispersion in the low frequency region is observed, followed by an almost frequency-independent behavior above 10 Hz. The decrease of ε′ can be attributed to electrode polarization and Maxwell-Wagner effect [85]. In the composite sample, we found higher values of ε′ at room temperature for f < 10 Hz. Moreover, we observed a severe temperature dependence of the dielectric constant that reach values almost one order of magnitude higher than in the reference sample. Regarding the imaginary part of the complex permittivity (Figure 6b,d), spectra of both specimens are characterized by a strong dispersion, as observed in ε′.

Hence, in order to investigate the relaxation processes occurring in the samples, we focused our attention on the loss tangent [85] defined as:

(4) $$tg\delta \left(\omega \right)=\frac{\varepsilon {\left(\omega \right)}^{\prime \prime }}{\varepsilon {\left(\omega \right)}^{\prime }}$$

**Figure 7:**
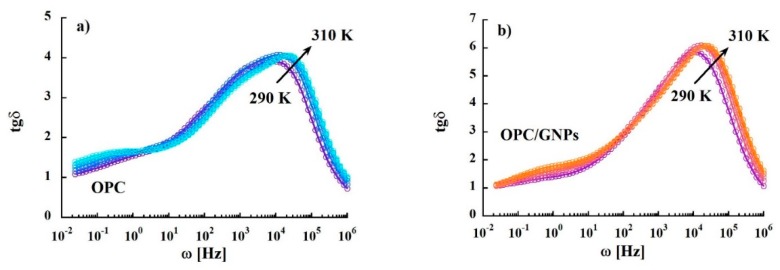
Variation of loss tangent in OPC (**a**) and OPC/GNPs (**b**) as a function of frequency in the temperature range 290–310 K.

In Figure 7a the spectra of the loss tangent of the reference, measured at different temperatures, are shown. In the whole temperature range the presence of two peaks is noticed. The most intense has the maximum centered at around 30 KHz and it does not show a strong dependence on temperature of the maximum position. On the other hand, the less intense peak at lower frequency is characterized by a clear dependence on the temperature of the peak maximum. Spectra of the loss tangent of the OPC/GNPs sample are shown in Figure 7b. The peak in the high frequency region is more intense than that found in OPC sample, even though the temperature dependence appears to be similar. On the other hand, the low frequency peak shows a different behavior when temperature is increased: The position of the maximum does not change with temperature, while the intensity increases.

**Figure 8:**
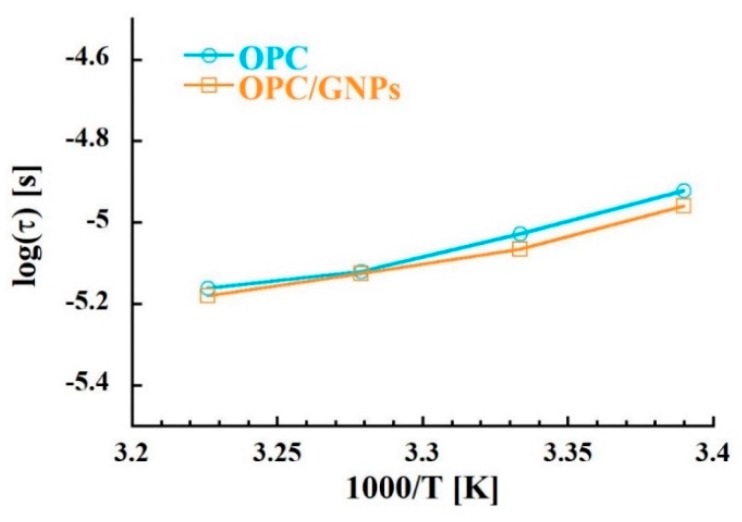
Temperature dependence of relaxation times extracted by the position of the peak maximum in the loss tangent.

Regarding the origin of those contributions, the peak at lower frequency appears where polarization effects were found both in the impedance response and in conductivity. Therefore, we believe that this contribution is given by electrode polarization or Maxwell-Wagner effect instead of some relaxation process. On the contrary, the intense peak of loss tangent appears in the frequency region where direct current is observed. A contribution with a similar weak temperature dependence was found at room temperature and at the same frequency range also in other porous materials [86,87,88]. In these works, the peak was related to the percolation of charge carriers through the porous network. To confirm this origin, we analyzed deeper the dielectric loss tangent spectra. In Figure 8 the relaxation times extracted from the position of the maximum of the peak are shown as a function of the inverse of temperature. In the composite specimen, a slight decrease of the relaxation times is observed. Furthermore, we observed a linear temperature dependence of log ($\tau_{0}$) typical of a simply thermal activated process.

Therefore, with the aim of obtaining information on the nature of the relaxation processes, the temperature dependence of *τ* values has been fitted with an Arrhenius law:

(5) $$\tau =\tau_{0}\exp(-\frac{E_{A}}{k_{B}T})$$

where *τ*_0_ is the pre-exponential factor the characteristic relaxation times of the process, *E_A_* is the activation energy, *k_B_* is the Boltzmann constant and *T* is the temperature. The resulting activation energy and pre-exponential factor parameters are shown in Table 4. First, we observed that the values of the pre-factor are larger than those typically observed for molecular vibrations ($\tau_{0}\approx$ 10^−14^–10^−12^ s). Moreover, the activation energies are similar to those found for the self-diffusion of ions through water in argillaceous rock porous network [89]. It is worth noting that the values of the activation energy calculated by the dielectric loss tangent correspond to those obtained by the direct current analysis indicating a correlation between these quantities. Therefore, we can associate the intense peak observed in dielectric loss tangent to the percolation of water molecules contributing to ion conductivity.

Finally, such a scenario is supported by modulus formalism analysis with complex modulus defined as:

(6) $$M^{*}=M^{\prime }+iM^{\prime \prime }=\frac{1}{\varepsilon^{*}}$$

**Figure 9:**
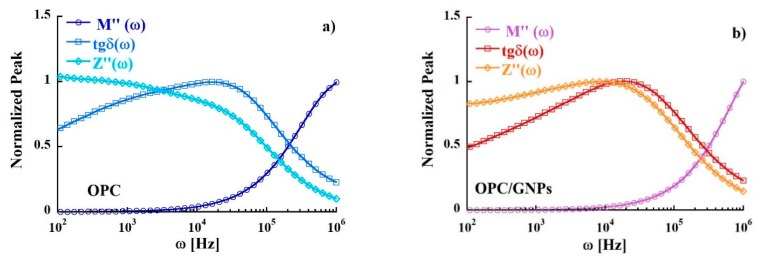
Comparison of tgδ, M″ and Z″ as a function of frequency at 300 K in OPC (**a**) and OPC/GNPs (**b**).

In Figure 9 the peaks of Z″, M″ and tgδ (normalized to the maximum value) are shown. We observed a significant mismatch between the position of the peak of the maximum in the imaginary part of electric modulus and the imaginary part of impedance. Such condition indicates a short-range nature of the motion of the charge carriers instead of long-range hopping mechanism [90].

Trying to elucidate how the presence of graphene nanoplatelets affect conductivity in cement paste, we summarize the main results of this work: First we observed an enhancement of the electrical properties in the composite by means of impedance response study, while dielectric response investigation proved that the main mechanism of charge transport is water percolation. At first sight, an increase of conductivity might be related to an increase of charge carriers in the composite, that is more water molecules in the specimen with GNPs. However, TG and DTG measurements showed that the samples have similar free water and bound water amount. Therefore, it is not possible to associate a raise of conductivity to an increase of charge carriers caused by GNPs presence. Consequently, the addition of graphene nanoplatelets must lead to some topological changes in the cement paste. This interpretation is supported by previous experimental works. In fact, it has been shown how the addition of graphene nanoplatelets lead to a refinement of the pore structure [43,91]. A small amount of GNPs reduces the percentage of macropores, even though the total porosity is slightly affected suggesting a broader distribution of small gel pores in the composite. Moreover, during the hydration process, C-S-H particles bonded to graphene flakes act as a nucleation site and promote the growth of C-S-H gel with higher crystallinity degree [92]. Under this scenario, we can explain the increase of ionic conductivity in relation with the enhancement of ordered C-S-H gel that promote a smoother diffusion of water molecules through the porous network. This would also explain the decrease of the energy activation of the relaxation process associated to water percolation and the slight smaller values of its relaxation times in OPC/GNPs sample.

## 4. Conclusions

With the aim of understanding the processes involved in conduction in cement-based materials, OPC cement paste and OPC/GNPs composite were studied by dielectric spectroscopy over a broad frequency range (10^−2^–10^6^ Hz) and at different temperatures. At low frequency, the enhancement of charge transport properties, reflected by the decrease of bulk resistivity and Z′, is observed once graphene nanoplatelets are added to the cement paste. Moreover, the electrical capacitance shows higher values in the composite over all the frequency range investigated. Finally, the effect of GNPs on the dielectric response of the system was also investigated. Regarding conductivity, larger values of direct current was found in OPC/GNPs. Information on the origin $\sigma_{DC}$ was extracted by analyzing its temperature dependence: *E_A_* and n parameters calculated for both samples suggest that the main contribution to direct current is given by ion diffusion. Moreover, at around ~10^4^ Hz, an intense peak is found in the loss tangent spectra. Previous dielectric studies on porous systems found a similar process related to water percolation through the pore network. The relaxation times associated to the maximum of this contribution were studied as a function of temperature: the calculated activation energy is close to that found for $\sigma_{DC}$ in the same frequency window and, therefore, these processes are correlated. Hence, we associated the intense peak observed in dielectric loss tangent to the percolation of water molecules contributing to ion conductivity. With respect to the role of the filler in the conduction enhancement, an increase of conductivity in the composite cannot be associated with an increase of charge carriers because TG and DTG measurements revealed that GNPs do not affect the water populations in the pore network. As graphene leads to a refinement of the pore, we propose that the enhancement of conductivity is mainly given by the ordered C-S-H gel structure growth around GNPs promoting the water diffusion and, therefore, resulting in an increase of conduction. It is clear that our results are calling for new experiments devoted to exploring the subtle changes provoked by GNP into the cementitious pore-network to favor the water percolation. In that sense, the combination of experiments and techniques like SANS [93,94], H-NMR [95,96], porosity measurements [97] can provide valuable help in understanding the conductivity of cementitious composites.

## Figures and Tables

**Table 1:** Free water, non-evaporable water cw% and portlandite content obtained by TGA analysis.

Sample	Free Water	Bound Water	Ca(OH)_2_ (%)
OPC	7	7.2	15
OPC/GNPs	6.5	7.5	15

**Table 2:** Parameters obtained from the Arrhenius equation applied to data in Figure 5.

	OPC			OPC/GNPs		
**T(K)**	**σ_DC_ (S/cm)**	**A**	**n**	**σ_DC_ (S/cm)**	**A**	**n**
**290**	3.9 × 10^−7^	3.11 × 10^−7^	0.17	1.8 × 10^−6^	5 × 10^−7^	0.17
**295**	6.0 × 10^−7^	3.6 × 10^−7^	0.17	2.4 × 10^−6^	6.1 × 10^−7^	0.17
**300**	7.0 × 10^−7^	4.1 × 10^−7^	0.17	2.9 × 10^−6^	6.9 × 10^−7^	0.17
**305**	7.6 × 10^−7^	4.6 × 10^−7^	0.17	3.2 × 10^−6^	7.7 × 10^−7^	0.17
**310**	8.5 × 10^−7^	4.7 × 10^−7^	0.17	3.6 × 10^−6^	8.3 × 10^−7^	0.17

**Table 3:** Activation Energy (*E*_A_) and pre-Exponential Factor (log [σ_0_]), which were obtained from the Arrhenius equation.

Sample	Log [σ_0_] (s)	E_A_ (eV)
OPC	−1.5	0.28
OPC/GNPs	−1.2	0.26

**Table 4:** Activation Energy (*E_A_*) and pre-Exponential Factor (log [*τ*_0_ (s)]) values obtained from the Arrhenius equation applied to data in Figure 8.

Sample	Log [*τ*_0_ (s)]	*E_A_* (eV)
OPC	−10	0.30
OPC/GNPs	−9.5	0.26
